# Karyotype analysis and sex determination in Australian Brush-turkeys (*Alectura lathami*)

**DOI:** 10.1371/journal.pone.0185014

**Published:** 2017-09-14

**Authors:** Madison T. Ortega, Dustin J. Foote, Nicholas Nees, Jason C. Erdmann, Charles D. Bangs, Cheryl S. Rosenfeld

**Affiliations:** 1 Bond Life Sciences Center, University of Missouri, Columbia, Missouri, United States of America; 2 Biomedical Sciences, University of Missouri, Columbia, Missouri, United States of America; 3 Sylvan Heights Bird Park, Scotland Neck, North Carolina, United States of America; 4 Department of Biology, East Carolina University, Greenville, North Carolina, United States of America; 5 Cytogenetics Laboratory, Stanford Health Care, Palo Alto, California, United States of America; 6 Thompson Center for Autism and Neurobehavioral Disorders, University of Missouri, Columbia, Missouri, United States of America; Leibniz-Institute of Freshwater Ecology and Inland Fisheries, GERMANY

## Abstract

Sexual differentiation across taxa may be due to genetic sex determination (GSD) and/or temperature sex determination (TSD). In many mammals, males are heterogametic (XY); whereas females are homogametic (XX). In most birds, the opposite is the case with females being heterogametic (ZW) and males the homogametic sex (ZZ). Many reptile species lack sex chromosomes, and instead, sexual differentiation is influenced by temperature with specific temperatures promoting males or females varying across species possessing this form of sexual differentiation, although TSD has recently been shown to override GSD in Australian central beaded dragons (*Pogona vitticeps*). There has been speculation that Australian Brush-turkeys (*Alectura lathami*) exhibit TSD alone and/or in combination with GSD. Thus, we sought to determine if this species possesses sex chromosomes. Blood was collected from one sexually mature female and two sexually mature males residing at Sylvan Heights Bird Park (SHBP) and shipped for karyotype analysis. Karyotype analysis revealed that contrary to speculation, Australian Brush-turkeys possess the classic avian ZW/ZZ sex chromosomes. It remains a possibility that a biased primary sex ratio of Australian Brush-turkeys might be influenced by maternal condition prior to ovulation that result in her laying predominantly Z- or W-bearing eggs and/or sex-biased mortality due to higher sensitivity of one sex in environmental conditions. A better understanding of how maternal and extrinsic factors might differentially modulate ovulation of Z- or W-bearing eggs and hatching of developing chicks possessing ZW or ZZ sex chromosomes could be essential in conservation strategies used to save endangered members of Megapodiidae.

## Introduction

Gonadal sexual differentiation during embryonic development may involve several genes. In mammals, these genes are for the most part located on sex chromosomes with females lacking male-promoting genes that reside on the Y chromosome. There are, however, notable exceptions as two spiny rat species, Amami spiny rat (*Tokudaia osimensis*) and Tokunoshima spiny rat (*T*. *tokunoshimensis*) that reside on two islands off the coast of Okinawa, Japan, with derived system of sex determination [1, 2]. Sexual differentiation in this species is likely directed by different dosages of genes residing on autosomal chromosomes. Recently, Monica Ward’s group generated transgenic mice lacking a Y chromosome [3]. In these animals, two transgenes, *Sox9* and *Eif2s3x*, compensated for the absence of Y-chromosome encoded genes that gave rise to males who could sire offspring.

In contrast to mammals where males are heterogametic (XY), females are heterogametic (ZW) in most birds. Thus, the female can influence the sex of her offspring by differentially laying Z- or W-bearing eggs. Reptiles may exhibit genetic sex determination (GSD) via sex chromosomes and/or temperature sex determination (TSD). It has recently been shown that in Australian central beaded dragons (*Pogona vitticeps*), which typically demonstrate GSD, individual sex can be overridden at high incubation temperatures that gives rise to sex-reversed female offspring [4]. It has been suggested that one avian species, the megapode bird, Australian Brush-turkey (*Alectura lathami*), may be unique in demonstrating TSD alone or in combination with GSD, similar to Australian central beaded dragons [5, 6]. Example male and female Australian Brush-turkeys are shown in S1 Fig. In this species, females engage in mate choice by observing male activity prior to copulation, and males can have several females laying eggs at a time. As newly hatched brush-turkey chicks are precocial, the female’s investment in them ends after the eggs are laid. After the eggs are laid, the male will monitor the temperature of the nest with his tongue and can adjust it by removal or addition of nesting material (S1 Fig and S1 Video- times 00:13, 00:17, and 00.22 seconds show this male behavior). Thus, the evolution of TSD in this species has been postulated. It remains to be determined though whether the resulting offspring sex ratio is due to incubation temperature and/or interaction with sex chromosomes. One report cited a “personal communication” as evidence that this species may have sex chromosomes [5]. However, evidence of such has not been reported to date. ZZ/ZW sex chromosomes have been described in other species within the family Megapodidae [7]. Thus, we sought to determine whether Australian Brush-turkeys possess heterogametic sex chromosomes.

## Materials and methods

### Life history of Australian Brush-turkeys included in the study

All three sampled Australian Brush-turkeys are captive born specimens currently house at Sylvan Heights Bird Park (SHBP) in North Carolina, USA. They are undoubtedly decedents from the nominate race, *A*. *l*. *lathami* [8] based on body size, iris/wattle color, and importation records. SHBP facility identification numbers are K1380 (male), K1878 (female), and K1379 (male). Blood was withdrawn on April 11^th^, 2017 as staff moved all three birds from their indoor wintering aviaries to the summer breeding aviary. K1380 (male) and K1878 (female) are shown in S1 Fig and S1 Video. These studies were approved by the ethics board at the SHBP.

### Blood collection

From the two males and one female, blood was collected from the basilic wing vein (located on the ventral surface of the proximal ulna) via an 18-gauge needle (Catalogue number: 305196, Becton, Dickinson and Company, Franklin Lakes, NJ) connected to a 3cc syringe (Catalogue number: 305196, Becton, Dickinson and Company). Avian blood sample collection protocol followed in accordance with Harrison’s Clinical Avian Medicine [9]. The samples were then placed vertical in a rack on ice and shipped overnight to the Cytogenetics Laboratory at Stanford Health Care in Palo Alto, CA, where the samples were immediately processed.

### Cytogenetic analysis

Using standard cytogenetic methodologies [10, 11], peripheral blood buffy coat obtained by centrifugation was inoculated into suspension culture using RPMI 1640 medium supplemented with 15% fetal bovine serum 50 μg/mL gentamycin sulfate and 2mM L-glutamine. Cultures were mitogenically stimulated with 1% Gibco phytohemagglutinin (Life Techonologies Corp, Carlsbad, CA) and pokeweed 10 μg/mL mitogen (Sigma, St. Louis, MO) and incubated at 37°C and 40°C. Cultures were harvested at 72 hours following a two hour mitotic arrest with 0.05 μg/mL Colcemid^®^ and one hour addition of 10 μg/mL ethidium bromide using standard methodologies of hypotonic shock with 0.075 M KCl and fixation with methanol:acetic acid fixative (3:1) [12, 13]. Metaphase preparations were made by dropping fixed cell suspension onto wet microscope slides, flooding with fixative and air-drying. Slides were aged at 90°C for 30 minutes and stained independently by trypsin/Giemsa G-banding and barium hydroxide C-banding [11, 12]. Metaphase cells were imaged and analyzed with an Olympus BX41 microscope (Olympus Corp., Center Valley, PA) 100x planapochromatic objective and Leica CytoVision image/karyotype system (Leica Microsystems Inc., Buffalo Grove, IL).

## Results

**Fig 1:**
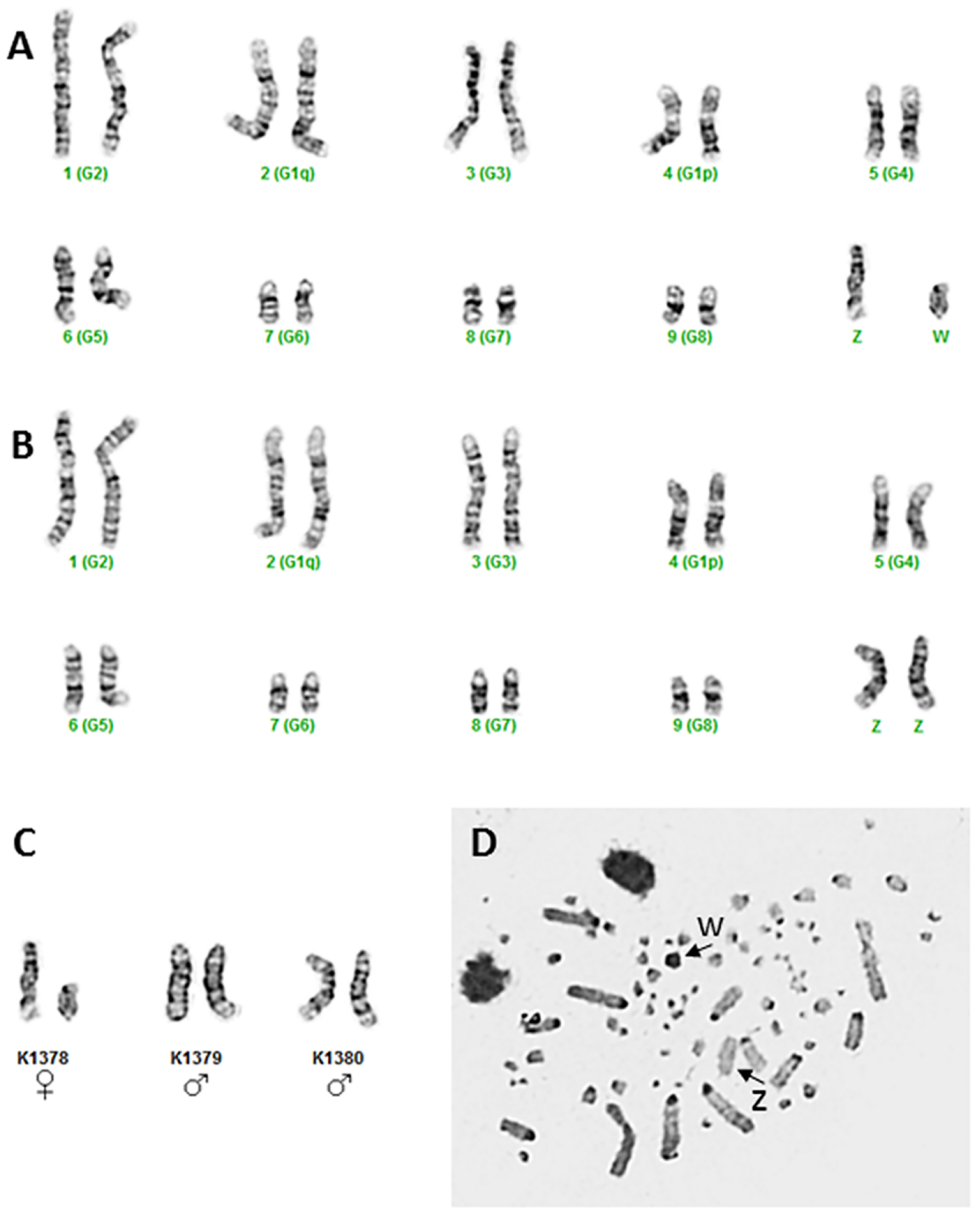
Karyotype results for Australian Brush-turkeys. G-band karyotype images without microchromosomes of A) female *A*. *lathami* demonstrating heterogametic ZW sex chromosomes and B) male *A*. *lathami* demonstrating homogametic ZZ sex chromosomes, C) G-band sex chromosomes of one female and two male *A*. *lathami*, and D) a C-band female metaphase image demonstrating weak to absent C-banding on the Z chromosome and complete C-banding on the W chromosome. For A and B, the numbers of corresponding chromosomes from the *G*. *gallus domesticus* karyotype are provided parenthetically.

G-banded chromosome analysis demonstrates an Australian Brush-turkey karyotype consisting of approximately 80 chromosomes. The karyotype is interpreted in reference to the standardized Domestic Chicken *(Gallus gallus domesticus)* [14] and other galliform lineage karyotypes [15, 16] as including 10 macrochromosome pairs and approximately sixty microchromosomes (Fig 1). Macrochromosomes include heteromorphic Z and W sex chromosomes. Imprecision regarding the exact chromosome number reflects the technical challenge of enumeration and classification of small microchromosomes in typical avian metaphase preparations. Based on G- and C-band analyses, chromosome #1 is morphologically sub-metacentric and chromosomes # 2 through #9 are telocentric. Any Z chromosome C-band is nearly indiscernible, however the chromosome overall appears morphologically telocentric. Based on C-band staining, the W chromosome is largely heterochromatic. Comparative G-band analysis indicates that the chromosome #1 common to other published galliform karyotypes represents a fusion of the Australian Brush-turkey chromosomes #2 and #4, where chromosome #2 corresponds to a common galliform #1 long arm and #4 corresponds to the galliform #1 short arm. Consequently, the Australian Brush-turkey chromosome #1 corresponds to the *G*. *galus domesticus* chromosome #2, etc.

## Discussion

The initial goal of the study was to confirm that Australian Brush-turkeys have sex chromosomes. Chromosome analysis demonstrates that in the limited sample size (two males and one female) Australian Brush-turkeys possess heteromorphic Z and W sex chromosomes consistent with other known galliform karyotypes. G-band analysis also indicates that the Australian Brush-turkey, as a representative megapode, has a karyotype distinct from other galliform lineages by virtue of two separate autosome pairs (#2 and #4) present in other galliform lineages as a single fused chromosome #1. Whether this represents an evolutionary process of chromosome fusion or fission is uncertain. One possibility is that this is a fission of a common ancestral chromosome #1 still extant in the other galliform lineages, or it may represent a fusion event occurring in an ancestral galliform subsequent to separation of the megapodes. Either scenario is consistent with current phylogeny of the Galliformes, which separates Megapodiidae, who likely originated during the Cretaceous period, ancestrally from other Galliformes that were derived during Tertiary period [17–20]. However, the possibility that this represents a fission event from another galliform lineage rather than an ancestral galliform cannot be ruled out based on the current data. It is interesting to note that chromosome fusion as a speciation-associated karyotypic phenomenon is well-documented in primates where there is fusion of the chimpanzee and bonobo (*Pan troglodytes*, *P*. *paniscus)* chromsomes #12 and #13 to form the human chromosome #2 [21]. When comparing the Z chromosomes of the Australian Brush-turkey and *G*. *gallus domesticus*, the centromeric regions appear similar in that they both lack a distinct centromere C-band unlike most autosomes in either karyotype. The main differences are morphologic: probable telocentric (Australian Brush-turkey) *vs*. sub-metacentric (*G*. *gallus domesticus*). Additionally, *G*. *gallus domesticus* has a heterochromatic region not present in the Australian Brush-turkey. An inversion and a heterochromatic addition would account for the altered *G*. *gallus domesticus* Z chromosome relative to the Z chromosome of the Australian Brush-turkey.

With the documentation that the Australian Brush-turkey possesses sex chromosomes, it opens up several potential avenues by which this species can affect offspring sex ratio. For instance, the final sex ratio of Australian Brush-turkeys might vary based on interactions of offspring sex and nest temperature (i.e. temperature-dependent sex-biased embryonic mortality), as suggested by other reports [5, 6, 22]. Developing males appear to be more vulnerable at higher incubation temperatures; whereas, lower incubation temperatures tends to be lethal to females [22]. It is not clear why these sex-differences exist and whether they might relate to genes expressed on the now identified sex chromosomes (ZW) within this species. Incubation temperature can vary the dry mass of the yolk-free body and residual yolk of hatchlings in this species with elevated temperatures giving rise to chicks with reduced yolk-free body mass and greater residual yolk mass than those incubated at lower temperatures [23].

In most mammalian species, who possess sex chromosomes, a variety of maternal-associated mechanisms exist that can result in skewed offspring sex ratios [24–30]. In birds, skewed offspring sex ratio can result due to differential embryonic survival. However, as the heterogametic sex, females are the sex determining parent, and it could be that maternal factors differentially influence ovulation of Z- or W-bearing eggs. This has shown to be the case in the endangered flightless parrot located in New Zealand, the Kakapo (*Strigops habroptila*). By provisioning the females with additional nutrient supplements prior to ovulation, researchers were able to generate male-biased chick sex ratios, and thus, sex allocation theory might have practical importance in helping to vary the number of males and females available for breeding in this endangered species [31]. Further, a lek mating system is present in Kakapos where the males gather and show-off to the females who then select their reproductive partners. Males in the best body condition are likely successful in obtaining the best “booming sites” and thereby attract a greater number of females.

Studies with other avian species, including the Superb Starlings (*Lamprotornis superbus*), Homing Pigeons (*Columba livia domestica*), Meadow Pipits (*Anthus pratensis*), Gouldin Finches (*Erythrura gouldiae*), Tree Swallows (*Tachycineta bicolor*), Blue Tits (*Cyanistes caeruleus*), Red-capped Robins (*Petroica goodenovii*), Common Starlings (*Sturnus vulgaris*), and Lesser Black-backed Gulls (*Larus fuscus*) strongly indicate that maternal condition and surrounding environment can result in offspring sex ratio adjustments [32–41]. This maternal-induced offspring sex ratio skewing could be due to selective laying of Z- or W-bearing eggs or sex dependent differences in deposition of yolk proteins, hormones, or other nutrient factors within the egg. Variation in yolk androgen content has been previously identified in Australian Brush-turkeys [42].

The current data provides definitive evidence that Australian Brush-turkeys possess sex chromosomes. Additionally, the potential fusion of autosomal pairs #2 and #4 of other Galliforms to form chromosome #1 in Australian Brush-turkeys is likely consistent with the previously identified earlier lineage of Megapodiidae relative to other Galliformes. While past studies have explored how adjustments in nest temperature by male Australian Brush-turkeys affects egg composition and offspring sex ratio, no studies to date have considered how maternal condition and environment might affect offspring sex ratio in this species. With the characterization of sex chromosomes in this species, it suggests that future studies should be directed at examining how maternal condition might influence laying of Z- or W-bearing eggs. Additionally, genes expressed from the Z- or W- chromosome may interact with egg composition or incubation temperature to result in sexually dimorphic differences in survival under various intrinsic and extrinsic environments. Thus, the current studies that have definitively identified sex chromosomes in Australian Brush-turkeys may open up new avenues in research to examine how maternal condition, sex-chromosome expressed genes, and embryonic environment, interact to modulate primary offspring sex ratio, as appears to be the case with Australian central beaded dragons, where TSD can seemingly override GSD [4]. The main mechanisms that can affect offspring sex ratio in Australian Brush-turkeys, and likely other avian species, are summarized in S2 Fig. A better understanding of these complex interactions in Australian Brush- turkeys and other avian species may be critical in breeding-strategies designed to alter offspring sex ratio in species already genetically bottlenecked and on the brink of extinction.

## Supporting information

S1 Fig Male and female Australian Brush-turkeys. Comparison of example breeding male (Panels A and B) with an example breeding female (C and D) reveals that when the male is in full breeding mode, his wattle enlarges and become bright red in color. However, the female wattle, which is smaller, remains the same color and size from season to season. Females tend to be smaller than males, and the plumage of males is slightly darker.
(TIF)

S2 Fig Diagram of all the potential mechanisms that can result in skewing of primary sex ratio in Australian Brush-turkeys. A) As the sex-determining parent, females can selectively lay Z- or W-bearing eggs. She can also alter in a sex-dependent manner the amount of yolk proteins, hormones, or other nutritional factors within the egg. B) The male can affect offspring sex ratio by adjusting the temperature of the nest that may favor the survival of one sex over the other. C) It is also possible that both parents can affect primary offspring sex ratio by the collective methods shown in panels A and B.
(TIF)

S1 Video This video demonstrates how a male Australian Brush-turkey constructs a nest out of various materials. He will then proceed to check the temperature of it with his tongue and alter the amount of nesting material based on the perceived temperature. This behavior is demonstrated at 00:13, 00:17, and 00:22 seconds in the video.
(MP4)
