# Supplementary figures and images for: Karyotype analysis and sex determination in Australian Brush-turkeys (Alectura lathami)
Source: PLoS One. 2017 Sep 14;12(9):e0185014. doi: 10.1371/journal.pone.0185014 (PMC5599057; doi:10.1371/journal.pone.0185014)

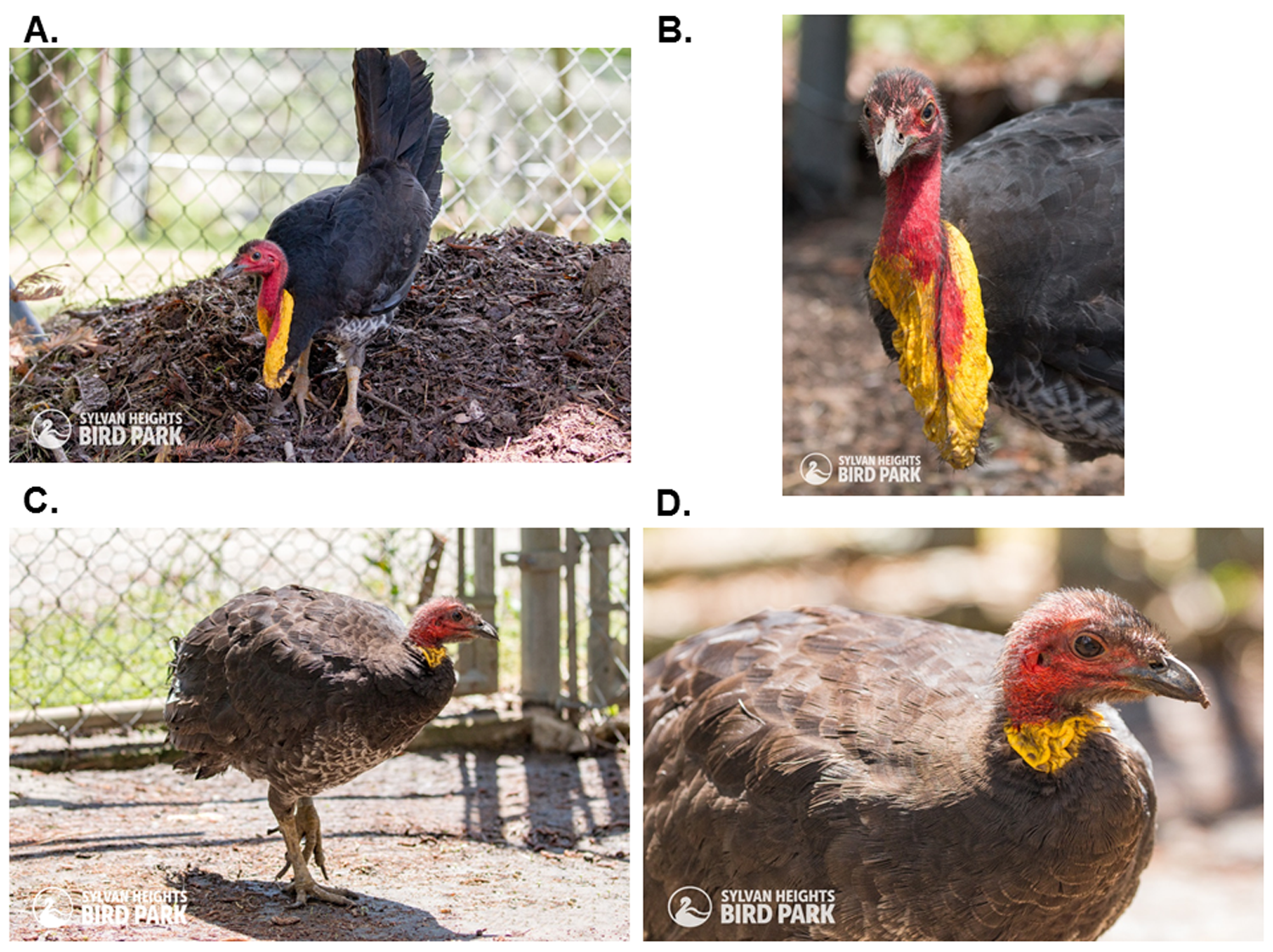

Supplement: S1 Fig — Comparison of example breeding male (Panels A and B) with an example breeding female (C and D) reveals that when the male is in full breeding mode, his wattle enlarges and become bright red in color. However, the female wattle, which is smaller, remains the same color and size from season to season. Females tend to be smaller than males, and the plumage of males is slightly darker. (TIF) [file pone.0185014.s001.tif]

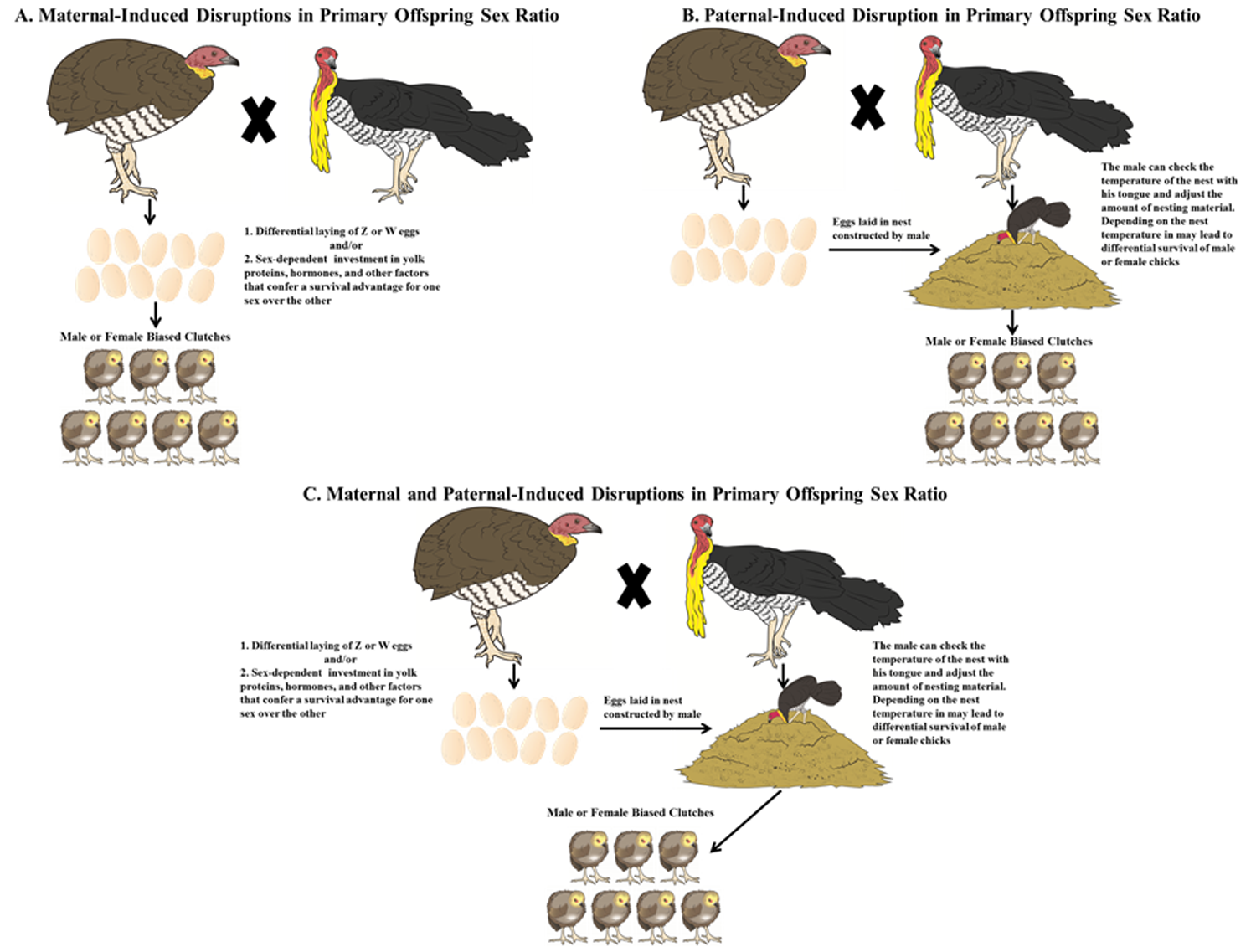

Supplement: S2 Fig — A) As the sex-determining parent, females can selectively lay Z- or W-bearing eggs. She can also alter in a sex-dependent manner the amount of yolk proteins, hormones, or other nutritional factors within the egg. B) The male can affect offspring sex ratio by adjusting the temperature of the nest that may favor the survival of one sex over the other. C) It is also possible that both parents can affect primary offspring sex ratio by the collective methods shown in panels A and B. (TIF) [file pone.0185014.s002.tif]
